# A global analysis of the complex landscape of isoforms and regulatory networks of p63 in human cells and tissues

**DOI:** 10.1186/s12864-015-1793-9

**Published:** 2015-08-07

**Authors:** Isha Sethi, Rose-Anne Romano, Christian Gluck, Kirsten Smalley, Borivoj Vojtesek, Michael J. Buck, Satrajit Sinha

**Affiliations:** 1 Department of Biochemistry, Center of Excellence in Bioinformatics and Life Sciences, State University of New York, 701 Ellicott Street, Buffalo, NY 14203 USA; 2 Department of Oral Biology, School of Dental Medicine, SUNY at Buffalo, Buffalo, NY 14214 USA; 3 Regional Centre for Applied Molecular Oncology, Masaryk Memorial Cancer Institute, Zluty kopec 7, Brno, 656 53 Czech Republic

**Keywords:** RNA-Seq, Keratinocytes, Transcription factor, Hierarchical clustering

## Abstract

**Background:**

The transcription factor p63 belongs to the p53/p63/p73 family and plays key functional roles during normal epithelial development and differentiation and in pathological states such as squamous cell carcinomas. The human *TP63* gene, located on chromosome 3q28 is driven by two promoters that generate the full-length transactivating (TA) and N-terminal truncated (ΔN) isoforms. Furthermore alternative splicing at the C-terminus gives rise to additional α, β, γ and likely several other minor variants. Teasing out the expression and biological function of each p63 variant has been both the focus of, and a cause for contention in the p63 field.

**Results:**

Here we have taken advantage of a burgeoning RNA-Seq based genomic data-sets to examine the global expression profiles of p63 isoforms across commonly utilized human cell-lines and major tissues and organs. Consistent with earlier studies, we find ΔNp63 transcripts, primarily that of the ΔNp63α isoforms, to be expressed in most cells of epithelial origin such as those of skin and oral tissues, mammary glands and squamous cell carcinomas. In contrast, TAp63 is not expressed in the majority of normal cell-types and tissues; rather it is selectively expressed at moderate to high levels in a subset of Burkitt’s and diffuse large B-cell lymphoma cell lines. We verify this differential expression pattern of p63 isoforms by Western blot analysis, using newly developed ΔN and TA specific antibodies. Furthermore using unsupervised clustering of human cell lines, tissues and organs, we show that ΔNp63 and TAp63 driven transcriptional networks involve very distinct sets of molecular players, which may underlie their different biological functions.

**Conclusions:**

In this study we report comprehensive and global expression profiles of p63 isoforms and their relationship to p53/p73 and other potential transcriptional co-regulators. We curate publicly available data generated in part by consortiums such as ENCODE, FANTOM and Human Protein Atlas to delineate the vastly different transcriptomic landscapes of ΔNp63 and TAp63. Our studies help not only in dispelling prevailing myths and controversies on p63 expression in commonly used human cell lines but also augur new isoform- and cell type-specific activities of p63.

**Electronic supplementary material:**

The online version of this article (doi:10.1186/s12864-015-1793-9) contains supplementary material, which is available to authorized users.

## Introduction

The transcriptional networks that govern temporally and spatially regulated gene expression programs are remarkably dynamic and complex [1]. One major factor that bestows this complexity is the incredible diversity of Transcription Factor (TF) isoforms that frequently arise from molecular events such as alternative splicing and promoter usage [2]. These isoforms, representing different protein products from the same gene, have distinct biological properties and thus can shape the transcriptional landscape of any cell in unique fashion. Hence any functional and deterministic studies of TFs should begin with careful parsing of the expression levels of its various isoforms across a wide-range of cell and tissue types. In the past, lack of large-scale expression data sets and appropriate tools such as isoform-specific antibodies have often hampered such studies and led to confusion and contradictory findings. This is quite evident in the case of the p53/p63/p73 family of proteins and is particularly true for p63 [3–6].

p63 is a developmentally important transcription factor involved in orchestrating a wide-ranging repertoire of biological functions such as cell fate determination, stem cell renewal, apoptosis and differentiation amongst others [7–12]. What complicates our understanding of p63 is that like other members of the p53 family, it is a structurally complex gene that generates multiple isoforms [7]. Thus the human *TP63* gene encodes for full-length transactivating (TA) and N-terminal truncated (ΔN) isoforms resulting from the usage of an upstream and an alternate intronic promoter, respectively. In addition, both TAp63 and ΔΝp63 transcripts undergo alternative splicing at the 3’ end resulting in at least three major C-terminal protein variants, termed α, β, and γ. These p63 isoforms share significant structural and functional homologies with p53 and p73 in the DNA-binding domain, which exhibit conservation of all essential DNA contact amino acid residues [13]. This similarity also extends to the transactivation and oliogomerization domains [3, 14]. In contrast, the α isoforms are unique to p63 and p73 in that they contain the sterile alpha motif (SAM) domain, which can act as a docking station for the formation of large protein complexes and a transcription inhibitory domain (TID). Not surprisingly, the complexities of the p63 isoforms weave a complicated functional interplay between themselves as well as within the extended network of the other two family members.

During the past several years, a number of experimental discoveries, driven primarily by the availability of isoform-specific knock out mouse models have been of immense value in improving our understanding of the physiological as well as pathological functions of p63 isoforms [15–20]. These studies have firmly established that ΔΝp63, in particular the ΔΝp63α isoforms are the predominant [21, 22] and most widely distributed proteins in many epithelial rich mouse tissues and organs and consequently are of the utmost functional relevance *in vivo*. Indeed, the bulk of the developmental role of p63 is shouldered by the ΔΝp63 isoforms. This is clearly evident in the phenotype of the ΔNp63-null animals, which show widespread defects of the skin, oral epithelium, mammary glands, limb and craniofacial regions—a developmental block that is quite similar to what has been reported for mice that lack all p63 isoforms [17–19, 23]. The specific role of TAp63 on the other hand has been mired in controversy, in part due to the conflicting and often unsubstantiated reports of its expression pattern under normal physiological conditions and its complex role in cancer [22, 24–26]. However, recent TAp63 specific knockouts have begun to shed some light and have revealed that this isoform plays only a minor role, if any, during normal epithelial development. The emerging evidence also suggests that TAp63 is induced in response to stress and under specific conditions, contributes to the maintenance of adult skin stem cells, serves as robust mediators of senescence and as regulators of lipid and glucose metabolism [16, 27].

The disparate role of p63 isoforms also extends to various types of human tumors, where there is aberrant expression of TAp63 and ΔNp63. In general the current consensus is that the ΔNp63 proteins act as oncogenes by antagonizing p53 and through other alternate p53-indepedent mechanisms, while TAp63 isoforms are tumor and metastatic suppressors—an observation supported by mouse models [20, 28–31]. However, results of studies from human tumors paints a complicated picture, with contradictory views in terms of the levels of expression of various p63 isoforms and its relation to clinical outcomes [28]. One major reason for such discrepancy is the fact that the p63 antibodies utilized in such studies do not distinguish between the TA and the ΔN isoforms. Moreover, these antibodies have been shown to cross-react with p73 [32]. Thus there is a dire need to carefully and methodically examine the expression profile of the various p63 isoforms, particularly in commonly used human cell lines, which over the years have been heavily exploited to test p63 function. With the advent of next generation sequencing techniques (such as RNA-Seq) and ever-increasing genomic datasets, it is now feasible to re-evaluate the p63 status in a global fashion.

Here, we have generated RNA-Seq datasets for several important HNSCC cell-lines, combined them with available datasets from the literature and the ENCODE project [33] to determine the expression profile of p63 isoforms. Our data reinforces the notion that ΔNp63 and TAp63 have very different expression patterns across a large number of human cell lines and tissues. In particular, we show that ΔNp63 mRNA is highly prevalent in epithelial cells from different tissues and epithelial tumor cell lines. Conversely, TAp63 expression is largely absent from commonly used cell lines, including a few that have been referenced in the literature and which have been utilized for TAp63-specific studies. Rather, we find that TAp63 is selectively expressed in B lymphoma cell lines. Importantly, we have confirmed our results by western blot analysis of a subset of selected human cell lines using newly developed ΔN/TA specific antibodies. Finally, we utilize unsupervised clustering analysis to generate network maps, which reveal distinct transcriptional co-regulators for ΔNp63 and TAp63, implying very different functional roles for the p63 isoforms.

## Results and discussion

### Transcriptomic map of human cells derived from existing and newly generated RNA-Seq datasets

With the increasing availability of expression data sets for a large number of human cell lines, it is possible to determine the expression profile of each p63 variant at a global level. Therefore, we mined publicly available RNA-Seq databases generated by the ENCODE project and existing literature to obtain transcriptomic maps of 34 commonly used and well characterized human cell types (Additional file 1: Table S1). Importantly, we chose representative human cells that corresponded to the three germ layers in terms of their likely developmental origins and also included primary, immortalized and tumor-derived types. In our search for suitable squamous cell carcinoma (SCC) cell lines for p63-centric studies, it was apparent that RNA-Seq expression profiles of SCCs were somewhat limited in the public domain. Given that p63 overexpression and/or genomic amplification is commonly observed in SCCs of the lung, esophagus and head and neck [28], we performed our own RNA-Seq based expression profiling of six SCC cell lines. These SCC cell types are different in both anatomical sites of origin and tumor grade (Additional file 1: Table S1). To ensure that the datasets were robust and not encumbered by shortcomings or limitations of any given computational method, the raw sequencing signal reads from these 40 human cell types were independently processed through two well-established RNA-Seq pipelines [34, 35] (see Methods). The gene expression estimates measured in fragments per kilobase of transcript per million (FPKM) obtained from the two different methods were highly concordant as evident by both similar FPKM values of TFs across individual cell-types and high correlation (median *r* = 0.9) across all cell-types (Additional file 2: Figure S1). For downstream analysis we utilized Analysis Pipeline 1 as our primary method to perform systematic examination and comparison of comprehensive gene expression patterns.

### TAp63 and ∆Np63 have distinct, non-overlapping expression patterns

**Fig 1:**
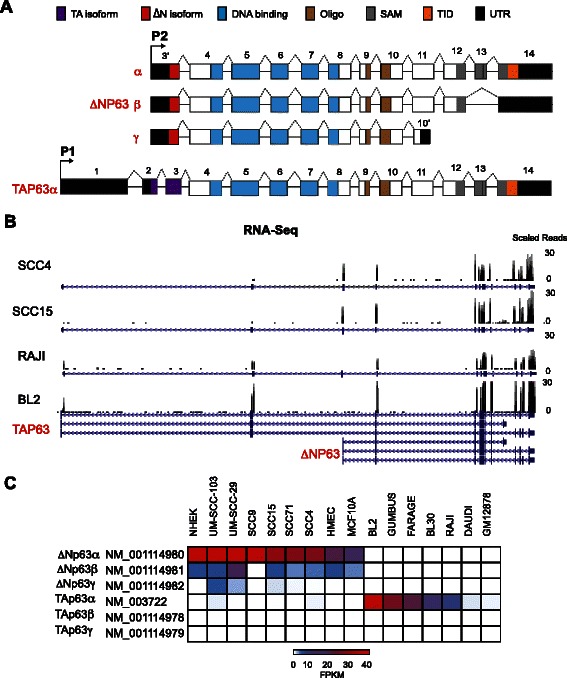
RNA-Seq reveals distinct expression patterns for TAp63 and ∆Np63. **a** Cartoon depicting ∆NP63 (α, β, γ) and TAp63α gene structure. TA β and γ (not shown) undergo similar alternative splicing to ∆NP63 (β, γ). TA, transactivation; Oligo, oligomerization; SAM, sterile alpha motif; TID, transactivation-inhibitory domain; UTR, untranslated region. **b** Snapshot from UCSC genome browser showing distribution of the aligned RNA-Seq reads at the *TP63* locus in representative epithelial cell-lines (Head and neck squamous cell carcinoma (SCC) cell-lines: SCC4 and SCC15) and Non-Hodgkin lymphomas (Burkitt lymphoma cell-lines: Raji and BL2). **c** Heatmap depicting the expression of p63 isoforms (average across replicates) in FPKM (fragments per kilobase of transcript per million) across representative p63 expressing cell-lines

The *TP63* gene generates full-length transactivating TA isoforms from an upstream promoter whereas an intronic promoter regulates the expression of the truncated ΔN transcripts (Fig. 1a, b). Using the transcriptomic profiles of the 40 human cell-types, we determined the relative distribution of TAp63 and ΔNp63 transcripts. Consistent with what has been previously reported in the literature, ΔNp63 transcripts were abundantly detected in keratinocytes derived from skin (NHEK and DK), oral tissue (OKF6) and primary (HMEC and HMEpC) as well as immortalized (MCF10A) epithelial cells derived from the mammary gland. In addition, ΔNp63 was also highly expressed in a wide range of squamous cell carcinomas (5 out of the 6 HNSCC cell-lines that were examined) (Fig. 1c, Additional file 3: Table S2). In contrast, the commonly used breast cancer cell lines expressed extremely low (less than five FPKM in MCF7) or undetectable (T47D, MDA-MB436 and MDA-MB231) levels of ΔNp63 (Additional file 3: Table S2). This result was surprising given that these commonly utilized breast cancer cell lines have served as valuable models for biochemical experiments to examine p63 function, as reported in several published studies [36–38]. Our analysis of the RNA-Seq data set also revealed that ΔNp63 transcripts were not detectable in a large number of cell types that represented non-epithelial developmental origins such as Normal Human Epidermal Melanocytes (NHEM), Normal Human lung fibroblasts (NHLF) or commonly used hematopoietic cancer cell lines (K562 or RAJI) (Additional file 3: Table S2).

Unlike ΔNp63, TAp63 expression levels were quite low across a wide spectrum of the cell lines examined. Indeed, a majority of the ΔNp63^+ve^ cell lines did not co-express TAp63 transcripts, although a few of the SCC cell lines and MCF7 showed very low levels of TAp63 mRNA expression. We also found that TAp63 was expressed at low levels in A549 cells, a human lung adenocarcinoma derived epithelial cell line, while the commonly used non-small cell lung cancer (NSCLC) cell line, H1299 did not express TAp63 (Additional file 3: Table S2). The lack of any TAp63 expression in H1299 as revealed by our analysis of the RNA-Seq data is particularly troubling given that there are published functional studies on endogenous TAp63 in this cell line [39, 40]. On the other hand, the RNA-Seq data fits well with an opposing view from other laboratories, which have primarily used the H1299 cell line as a p63-null model system to examine the consequences of addition of exogenous p63 [32, 41]. Among the likely explanations for this discordant result are mistaken cell identity or differences in specific growth conditions for H1299 cells. These conflicting results highlight the importance of careful evaluation of p63 expression levels by deep sequencing and when possible, verification of the transcript levels by independent and sensitive experiments such as quantitative RT-PCR (qRT-PCR) before choosing the appropriate cell line for studies on p63. Towards this end, we examined representative TAp63 and ΔNp63 expressing cell lines and performed isoform-specific qRT-PCR (Additional file 4: Figure S2). Our studies revealed that at least in these selected cell lines, the qRT-PCR based expression pattern of the N-terminal variants of p63 matched quite well with the corresponding data from RNA-Seq experiments, although the range of expression was noticeably pronounced as can be expected from the higher sensitivity of the PCR based approach.

Interestingly, instead of epithelial cell lines, TAp63 exhibited a wide range of expression levels in Burkitts Lymphomas (BL) cell-lines, with relatively strong in a few (BL2, GUMBUS, FARAGE, BL30), moderate to low in others (RAJI and DAUDI) and undetectable in another subset (BL70 and RAMOS for e.g.). Low levels of TAp63 were also observed in GM12878, a lymphoblastoid ENCODE Tier one cell line that has served as a workhorse for many of the genomic projects (Fig. 1c). Collectively our analysis revealed a complex expression pattern for the two major p63 isoforms and established that TAp63 and ΔNp63 have very different expression profile with ΔNp63 mainly expressed in cells of ectodermal lineage, whereas TAp63 is mostly restricted to cells of mesodermal origin.

One major caveat to the RNA-Seq based expression analysis is that it often exemplifies only a snapshot of a specific state of the cells. Furthermore, the data can be heavily skewed by various extraneous factors such as cell passage number, growth conditions and state of confluency among others. Thus it is likely that the final verdict on the expression (or lack there of) of p63 isoforms in a particular cell type will require comprehensive experiments that cover a wide range of physiological conditions. A case in point is the differentiation program of keratinocytes, which can be well mimicked in cell culture conditions. One prevailing notion in the field is that while ΔNp63 are the predominant isoforms in basal proliferating keratinocytes, differentiation of the keratinocytes is accompanied by a switch from ΔNp63 to TAp63 [42, 43]. To test this hypothesis, we examined RNA-Seq data obtained from human keratinocytes at different time points (DK0, DK3 and DK6) of the differentiation program [44]. This analysis showed that although there was a significant diminution in the expression levels of ΔNp63 transcripts with differentiation, surprisingly under no conditions was TAp63 expression detected (Additional file 5: Figure S3)—a finding that corroborates well with the results of RNA polymerase II ChIP experiments on the p63 TA and ΔN promoters [45].

Another factor that can influence the outcome of RNA-Seq based transcriptomic studies is the depth of sequencing coverage [46]. Indeed biological samples often have different numbers of transcribed genes/transcripts, different degree of transcriptome complexity and a dynamic distribution of expression levels for transcripts–all of which can make data interpretation quite challenging. Hence, it is possible that some of the p63 isoform expression data derived from the RNA-Seq experiments as described here, could in part be tinged with effects from over or under sequencing and thus might not be completely accurate in the biological context. We have tried to minimize this limitation by ensuring that most of the samples that were included were sequenced to a reasonable depth and wherever possible, replicates were analyzed. A complementary approach to test if a specific gene is indeed expressed or silent in a particular cell type is to probe chromatin accessibility data such as those generated from DNase-Seq experiments [47]. Hence, we examined the chromatin architecture of the two primary p63 promoters across three disparate cell types–human epidermal keratinocytes (NHEK), B cells (GM12878) and mammary adenocarcinoma (MCF7) for which DNase-Seq data was publicly available. As shown in Additional file 6: Figure S4, NHEK cells showed a clear DNase hypersensitive mark selectively at the ΔNp63 promoter but not at the TAp63 promoter whereas GM12878 cells displayed a diametrically opposite pattern. Therefore this data would suggest that the ΔN and TA promoters are open and active in keratinocytes and GM12878 respectively, in good agreement with the expression data from RNA-Seq. Interestingly, according to the DNase-Seq data, both the TA and ΔN promoters were inaccessible in MCF7 cells–this may very well account for the very low levels of TAp63 and ΔNp63 transcripts in this cell type.

### Relative abundance of the alternatively spliced isoforms of p63

Although RNA-Seq has rapidly become the method of choice for global transcriptomic studies, proper assembly, identification and expression quantification of gene transcripts remains a challenging process that is heavily dependent both on the computational software and the reference transcript annotation file used for genome-guided assembly [48]. Given the known complexity and extensive alternative splicing of the *TP63* gene we analyzed all RNA-Seq datasets using two pipelines, implementing different transcript reconstruction methods and annotation files (see Methods).

**Fig 2:**
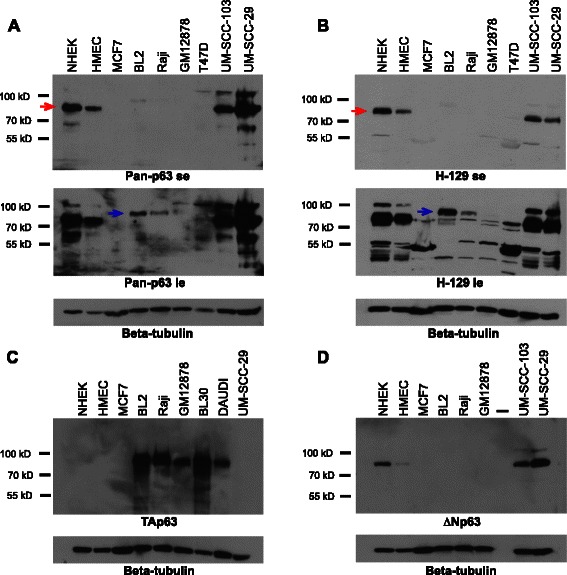
Protein expression profiles of p63 isoforms. Western blot analysis of whole cell extracts demonstrates p63 expression using (**a**) a pan-p63 antibody (**b**) and an alpha specific antibody (H-129). TAp63 and ∆Np63 isoform specific expression is shown in panels (**c**) and (**d**) respectively. Blue and red arrows mark the TAp63 and ∆Np63 protein bands, respectively. Beta-tubulin serves as a loading control. se: short exposure and le:long exposure

We ran the first analysis pipeline with the goal to examine the six major p63 isoforms that are annotated and well-defined in the literature (TAp63α, β, and γ, ΔNp63α, β, and γ) as well as to detect novel transcripts. We found that the longest C-terminal α variant was the predominant isoform expressed across all p63 positive cell-lines, irrespective of their source. Thus in all normal and tumorigenic cell lines, in which the ΔNp63 transcripts were detectable at appreciable levels, ΔNp63α was several log fold higher than ΔNp63β and ΔNp63γ. Of the two shorter isoforms, ΔNp63β displayed a relatively widespread distribution as evident from its low to modest expression (0–15 FPKM) in 11 out of 15 cell-lines, whereas ΔNp63γ on the other hand was detected only at low levels (<5 FPKM) in a small subset of HNSCC cell lines (Additional file 3: Table S2). Interestingly ΔNp63β levels remained consistent in differentiating keratinocytes unlike the ΔNp63α isoform (Additional file 4: Figure S2). The dynamic range of expression of the ΔNp63 isoforms suggests that there might exist a distinct functional role for each isoform, especially the β isoform. In most TAp63 expressing cell-lines, only the TAp63α isoform was detected at appreciable levels (Fig. 2). It is important to note that in oocytes, where TAp63α is highly expressed, it is thought to exist in an inactive conformation due to complex domain-domain interactions [49, 50]. Whether similar structural mechanism of TAp63α inhibition also operates in BL cells is an interesting question that needs to be addressed. Interestingly, a novel p63 isoform, TAp63δ was identified in the GUMBUS BL cell-line by the *de novo* method (as implemented in Analysis Pipeline 1), albeit at very low levels (data not shown). This is a shorter p63 isoform that is generated by exon skipping in the 3’ end and was only recently discovered [51].

Using a parallel approach we next examined the RNA-Seq datasets using Analysis pipeline 2 (see Methods) and employing a more comprehensive annotation library from Ensembl (contains both known and predicted p63 isoforms). We found that the expression estimates for major known p63 isoforms, i.e. ΔN (α, β and γ) and TAp63α were robust and their relative distribution levels and patterns were the same across the two methods. TAp63β and TAp63γ were not detected whereas TAp63δ was confirmed to be expressed in GUMBUS cells (similar to Analysis pipeline 1) and additionally detected at very low levels in FARAGE cells (Additional file 7: Figure S5). Interestingly, this analysis revealed several additional ΔN C-terminal variants, the most prominent being ΔNp63αΔ4, a relatively close cousin of ΔNp63α. This transcript is predicted to generate just four amino acids smaller protein product compared to ΔNp63α and has been reported to be expressed in mouse skin [21]. ΔNp63αΔ4 was present at moderate to high levels in a number of ΔNp63 positive cell-lines including keratinocytes, mammary epithelial and HNSCC cells. On the other hand a few novel ΔN isoforms such as ΔNp63ε and ENST00000434928 were detected only in HMEC cells at low levels. Whether these isoforms have a mammary epithelial cell specific function and/or their detection is merely a consequence of the high sequencing depth (~250 million reads) of HMEC RNA-Seq datasets needs to be further probed (Additional file 7: Figure S5). It is possible that the full spectrum of C-terminal variants for the TA isoform was not revealed by both methods due to the fact that the TA promoter is less active than the intronic ΔN promoter in most cell lines and under physiological conditions.

Overall our analysis suggests that even though both ΔN and TA transcripts undergo complex alternative splicing events, the major C-terminal variant expressed in most cell lines is the α isoform and that minor transcripts representing various splicing products can often be unearthed using the power of deep sequencing and sophisticated computational tools. We suspect though that within the biological confines of any given cell, the bulk of the p63 functional activity is driven by the major α isoform, while the minor isoforms perhaps represent the misfiring or over-activity of the splicing machinery. This is in agreement with the emerging general view that in a given condition, the transcriptome from protein coding loci is dominated by one major transcript per gene and often that same transcript is expressed across many cell-types [52]. Further support for the biological importance of the p63α isoform comes from recently generated mouse knockouts, which demonstrates that the C-terminus is vital to p63 function during embryonic development [53]. Similar *in vivo* biochemical studies for the p63β and γ as well as other minor isoforms will shed light on their functional significance.

### Detection of TAp63 and ΔNp63 proteins using a panel of specific antibodies

While the RNA-Seq data provides a global first-hand view of the repertoire of p63 transcripts in various cell types, in most cases there have been no careful follow-up studies to examine the corresponding protein products. One major problem that has contributed to the dearth of such protein-based studies is the lack of appropriate tools, specifically of validated isoform-specific antibodies. This has led to several unproven and likely erroneous assumptions in the field regarding the expression and stability of the p63 isoforms. Indeed, one such often-repeated and widely prevalent notion is that p63 isoforms may have inherently different stability and posttranscriptional regulations with TAp63 proteins being particularly susceptible to degradation and hence undetectable by western blot analysis.

Recently a panel of isoform-specific and pan anti-p63 antibodies were generated to address this concern [54]. Utilizing these antibodies we performed western blot experiments on a selected panel of cells for which the RNA-Seq data was available. As shown in Fig. 2, expression of various p63 protein isoforms was robustly detected in several of the cell lines tested. This is in good agreement with the corresponding mRNA level as determined by RNA-Seq analysis. Strong expression of ΔNp63 was observed in NHEK and HMEC cells as well as two representative SCC cell lines, UM-SCC-29 and UM-SCC-103. The expected size of ~80 kD corresponded to the ΔNp63α isoform. In contrast, a higher migrating band of ~85-90 kD was observed in BL2, RAJI and GM1278 cells which represent TAp63α. Western blotting with a commercial anti-p63 antibody, H-129, which specifically recognizes the α isoforms, confirmed this distinct and non-overlapping expression pattern of the α isoform of ΔNp63 and TAp63 in these cells. Furthermore it was also apparent that the expression of TAp63 in lymphoblast cell lines was considerably lower than the abundant levels of ΔNp63 proteins in epithelial cells, as evident by data from a longer exposure (see Fig. 2a and b lower panels). Next we utilized TA and ΔN-specific antibodies that have been well characterized in terms of their efficacy and specificity [54]. As shown in Fig. 2c, anti-TAp63 antibodies detected a strong band in five representative lymphoma cell lines with absolutely no detectable expression in epithelial cells. This experiment also revealed that the exquisite sensitivity of the monoclonal antibodies makes it feasible to detect low levels of TAp63 proteins even in cell lines that express very low mRNA levels (<1 FPKM in GM12878 for e.g.). In contrast, a band corresponding to ΔNp63α was clearly evident with anti-ΔNp63 antibodies in the representative epithelial cells with the highest expression levels in NHEK and SCC cells (Fig. 2d). It is also important to note that under these western blot conditions, we could not detect the less abundant isoforms of p63, such as β and γ - this observation is in good agreement with the analysis of the RNA-Seq data.

### Expression pattern of p63 family members

The availability of the RNA-Seq data allowed us to examine another important facet of p63 biology that is important but not as well appreciated. Given the structural similarities between p63, p53 and p73 [55], their DNA-binding affinity towards similar DNA sequences and the proclivity to heterodimerize, it is likely that the biological activity of p63 in a specific cell type will be greatly influenced by the presence or absence of the other two family members. Hence it is important to examine the relative expression levels of p53 and p73 in relation to ΔNp63 and TAp63. Of these two family members, p73 was the least abundant and showed negligible to low expression (<5 FPKM) across all of the 40 cell-types (Additional file 8: Figure S6). This is an expected finding, as p73 is important in neuronal development [56, 57] and neural cell-types were not represented in our dataset. In contrast p53 was moderately expressed across most of the cell-types. Interestingly, p53 mRNA levels were preferentially higher in breast adenocarcinomas (no expression of p63) and in NonHodgkin’s lymphomas (TAp63 expressing cell-lines) (Additional file 8: Figure S6). At this stage, it is difficult to ascertain if there is any significance to this finding. Another caveat to this interesting p53/TAp63 correlation is the fact that the p53 status is quite varied across the different cell-types ranging from wild-type, mutant to null. As expected, most of the cancer cell-lines contained a missense or small frameshift mutation in p53 (Additional file 1: Table S1). Since mutant p53 can form heterotetramers with p63 and studies have suggested that its gain-of-function properties can be attributed in part to its interaction with p63 [58], the overall biochemical attributes of p63 are likely to be influenced by the p53 expression and mutation status in a specific cell type.

### Meta-analysis of RNA-Seq expression data reveals transcriptional co-regulators of p63

**Fig 3:**
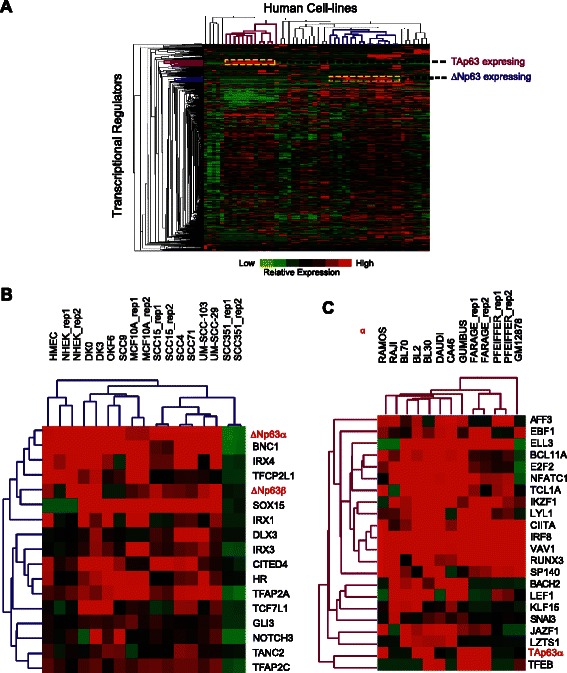
Transcription Factors predicted to coordinate with ΔNp63 and TAp63 based on expression profiles (**a**) Heatmap depicts hierarchical clustering of fold change in expression (over median) of 867 TFs across 40 cell-types (54 experiments). Trimmed dendrogram (correlation > = 0.6) highlights the transcriptional regulators with similar expression pattern to (**b**) ΔNp63 and (**c**) TAp63, across a subset of cell-lines (depicted by yellow dotted rectangle). The specific p63 isoforms are shown in red

Cooperative interactions between TFs constitute an important and indispensable regulatory force that drives tissue-specific gene expression programs. Hence, master regulatory factors such as p63 that play crucial roles in developmental and cell fate decisions are likely to be associated with an expansive repertoire of TFs. Presumably, these p63-linked TFs act in a concerted fashion to modulate the p63 biological output. One obvious corollary to this idea is the possibility that such co-regulators within the p63 network are likely to exhibit similar patterns of expression across different cell types. To this end, we examined expression datasets detailing the relative abundance of a large number of human TFs across 40 normal and cancer cell types. Given the distinct differences in expression patterns of ΔN and TA, we incorporated transcript-specific data information for p63 isoforms in our analysis of the expression dataset. The dataset was also filtered to remove both highly expressed ubiquitous TFs and those with less abundant transcripts (<5 FPKM in at least 1 cell-type). As ΔNp63γ and TAp63 (β, γ) isoforms are expressed at low levels in most of the human cells, they were not represented in this analysis. Unsupervised hierarchical clustering across both genes (867 differentially expressed TFs) and experiments (52 RNA-Seq data sets) revealed quite interesting expression patterns (Fig. 3a). Interestingly, cell-lines appeared to cluster according to the germ layer of origin and their physiological functions, irrespective of the karyotype (normal vs. cancer). Replicates (as represented by MCF7_rep1, MCF7_rep2) and cell-lines that were from identical sources (such as NHEK, DK0) usually clustered together, further ensuring that the method was unbiased to data originating from different sources (Additional file 9: Figure S7).

The dendrogram of the 867 differentially expressed TFs revealed those that are most closely associated (at correlation > = 0.6) with ΔNp63 and TAp63 based on their expression pattern. As shown in Fig. 3, ΔNp63 and TA clustered with distinct families of TFs. Importantly, as revealed by this clustering analysis, many of the likely transcriptional co-regulators of ΔNp63; TFAP2A, TFAP2C, NOTCH3 and DLX3 (Fig. 3b) are known and established players in the p63-related transcriptional circuitry and/or biological activity in skin keratinocytes [17, 59–61]. Similar results were also obtained from a recent study, which showed AP2 as a likely transcriptional co-regulator of p63 based on TF motif enrichment and overlapping in-vivo binding profiles at p63 target sites in NHEK [59, 62]. On the other hand, our analysis unearthed several novel players including members of the IRX family, SOX15 and CITED4 for which there is no published link with p63 (Fig. 3b). As expected from the virtually non-overlapping expression pattern of the two major p63 isoforms, TAp63 clustered with a very different group of TFs including LEF1, KLF15, IRF8, RUNX3–interestingly some of these have been shown to be associated with the p63 network and/or play a role in B-lymphomas [63–65] (Fig. 3c).

**Fig 4:**
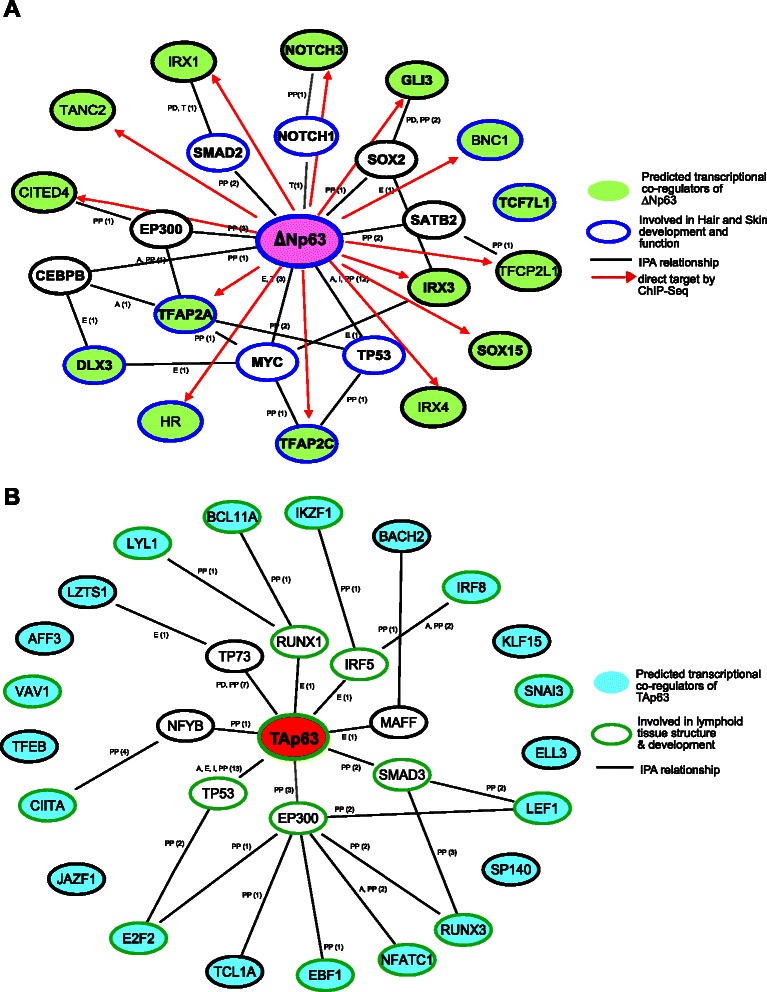
ΔNp63 and TAp63 TF networks. (**a**) Interaction Network for ΔNp63. Green molecules denote predicted co-regulators. Red arrows represent TFs that are likely direct targets of p63 as determined by overlay with ChIP-Seq information. Molecules with blue boundaries are those involved in Hair and Skin development and function. (**b**) Interaction Network of TAp63. Molecules denoted in blue are the predicted co-regulators. Molecules with green boundaries are involved in lymphoid tissue structure and development. The interaction networks (black lines connecting molecules) were generated by QIAGEN’s Ingenuity Pathway Analysis. The specific type of evidence in literature is denoted by–PP: Protein-Protein interaction, A: Activation, I: Inhibition, T: Transcription, E: Expression, PD: Protein-DNA interaction

In order to gain a deeper understanding of the relationships between ΔNp63 and TAp63 and the transcriptional co-regulators that are likely to be biologically relevant, we used Ingenuity Pathway Analysis (IPA) to generate network maps (Fig. 4a and b). IPA primarily uses experimental evidence as documented in the literature to define the pathway (shown by black lines) between two molecules. The notification next to the relationship indicates the type of evidence that is described in the literature (PP: Protein-Protein interaction, A: Activation, I: Inhibition, T: Transcription, E: Expression, PD: Protein-DNA interaction). We overlaid the maps with Functions and Disease data from IPA that uses Gene Set Enrichment Analysis (GSEA) to find the significant biological functions regulated by a group of factors. Most of the likely transcriptional co-regulators (green molecules) that clustered with ΔNp63 were deemed to be linked within a ΔNp63 specific network, and importantly are involved in biological functions that include hair and skin development and function (*p* value: 5.46E-14–5.56E-6) (molecules with blue boundaries) (Fig. 4a, Additional file 10: Table S3). Based on this analysis we postulate that other novel TFs that have similar expression pattern to ΔNp63 (such as IRX family members and SOX15) are likely to be an integral part of the p63 transcriptional network with a hitherto undiscovered functional role in epithelial biology. Interestingly by overlaying the ΔNp63 network with p63 ChIP-Seq data (red arrows) from primary human keratinocytes [66], we found that 8 out of the 15 predicted transcriptional co-regulators are also direct targets of p63 (Fig. 4a). This strongly raises the possibility that ΔNp63 is positioned in a central hub in the transcriptional circuitry of many human cells where it directs gene expression in a coordinated fashion with other TFs.

Similar IPA analysis elucidated the TAp63 centric network and unearthed interesting relationships between TAp63 and its predicted co-regulators (blue molecules) in human cells. Many of these factors (12 out of 21) are thought to be involved in a distinct biological function, that of lymphoid tissue structure and development (*p* value: 5.17E-20–4.75E-5) (molecules marked with blue boundaries) (Fig. 4b). Indeed, aberrant expression and activity of TAp63 [67] together with some of these co-expressing TFs such as LEF1, BCL1A, RUNX3, IRF8, have been linked to lymphomas [64, 68, 69]. Based on this expression-based data, it is tempting to speculate that the TAp63 network might constitute an important regulatory module in lymphomas. Whether like ΔNp63, TAp63 also directly regulates many of the co-expressed TFs will necessitate future studies such as ChIP-seq with anti-TAp63 antibodies in TAp63-enriched lymphoma cell lines such as BL2 and BL30.

### Expression pattern of p63 isoforms in human tissues and organs

**Fig 5:**
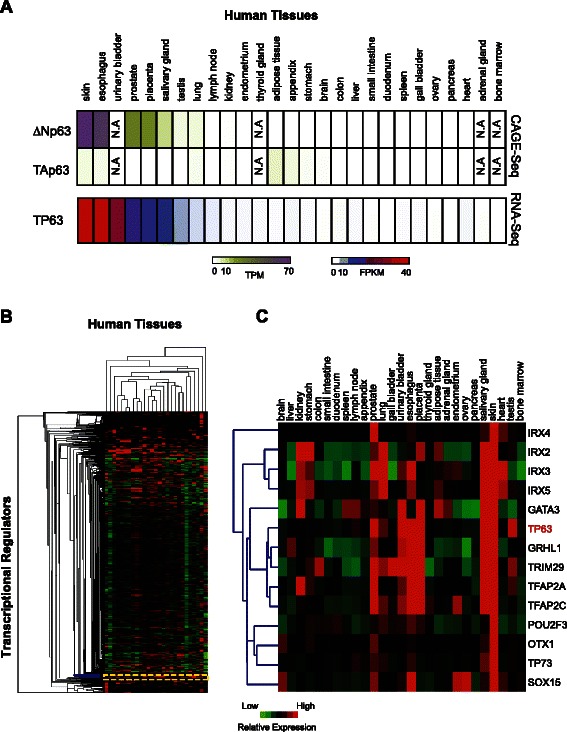
Predicted transcriptional co-regulators of ΔNp63 in human tissues and organs (**a**) Heatmap depicting transcript abundances of p63 across major human tissues and organs using CAGE-Seq and RNA-Seq (as obtained from the FANTOM database and the Human Protein Atlas). TPM: transcripts per million; FPKM: fragments per kilobase of transcript per million. (**b**) Hierarchical clustering of fold change in RNA-Seq expression (over median) of 867 TFs (rows) across 27 major tissues and organs, (**c**) Close-up shows a trimmed dendrogram (correlation > = 0.65) depicting the TFs with most similar expression pattern to p63 (shown in red)

Thus far all of our analysis was based on RNA-Seq expression data from cell-lines, which may not reflect the physiological state of human tissues. In order to determine whether the cell culture based results were indicative of p63 expression *in vivo*, we next examined the RNA-Seq data and Cap Analysis of Gene Expression (CAGE) datasets that have been recently generated by the Human Protein Atlas Project [70] and the FANTOM Consortium [71], respectively. The CAGE information, which provides a readout of the human transcription start site activity, allowed us to specifically examine the ΔNp63 and TAp63 promoter in a number of human tissues and organs. On the other hand, the RNA-Seq data were pre-processed that provided overall p63 expression levels without any isoform-specific details. The RNA-Seq and CAGE data sets showed a congruous pattern and reaffirmed the notion that similar to what was observed for cell lines, ΔNp63 is the predominant isoform in human tissues and organs, particularly in those that are epithelial-enriched. Indeed ΔNp63 was highly expressed in skin, esophagus, prostate and other epithelial-enriched tissues, whereas TAp63 was weakly expressed or absent in most of the examined tissues with low expression in a few tissues and cell types such as adipocytes (Fig. 5a).

The availability of human tissue RNA-Seq datasets also allowed us to perform unsupervised hierarchical clustering using Pearson correlation distance metric similar, to what was done with the cell-line datasets (Fig. 5b). The set of TFs that cluster with p63 in human tissues (Fig. 5c) match well with the predicted transcriptional co-regulators of ΔNp63 based on cluster analysis of the cell-line expression datasets (see Fig. 3b). Thus a number of TFs, in particular AP2 and IRX family members and SOX15 have a similar expression pattern to p63 (ΔNp63) across both tissues and cell-lines. It is also important to note that a subset of these p63 co-factors were also uncovered in a similar clustering analysis of mouse tissues [21] suggesting that there exists an evolutionarily conserved transcriptional network that is anchored by ΔNp63. In contrast, we did not find any TAp63 associated TFs that clustered with p63 in the tissue expression dataset. This is an expected finding because of low TAp63 mRNA levels across the spectrum of the human tissues. These findings collectively reinforce the prevailing notion that ΔNp63 is the predominantly expressing isoform and hence functionally relevant in most human tissues and organs, as is the well-documented case in mouse.

## Conclusions

Our comprehensive analysis of the isoform diversity and dynamic expression pattern of p63 in various human cell lines and tissues as described in this report, highlights the power of emerging genomics tools to generate meaningful data and testable hypotheses. We show that the two major isoforms of p63, ΔNp63 and TAp63 have their own distinct expression profile, which is like to dictate their unique biological activities and functional partnerships. In future, in depth examination of additional human cell lines and tissues, representing a wide range of physiological and pathological states will surely offer new insights into the p63 family of factors and their broad transcriptional network.

## Materials and methods

### Cell lines

Human HNSCC cell-lines SCC15, SCC351, SCC4, SCC71, UM-SCC-29 and UM-SCC-103 were grown in high glucose (25 mM) DMEM media containing 1 % penicillin/Streptomycin, 4 mM L-Glutamine and 10 % fetal bovine serum. For UM-SCC-29 and UM-SCC-103 (obtained from University of Michigan [72, 73]) the media was also supplemented with 1 % non-essential amino acids. Lymphoma cell lines BL2, BL30, Raji and Daudi were grown in RPMI 1640 media containing 10 % fetal bovine serum, 1 % penicillin/streptomycin and 1 % GlutaMAX. Breast cancer cell lines MCF7 was grown in high glucose (25 mM) DMEM media containing 1 % penicillin/Streptomycin and 10 % fetal bovine serum, whereas T47D cells were grown in RPMI media supplemented with 1 % penicillin/Streptomycin and 10 % fetal bovine serum. NHEK and HMEC cells were obtained from Lonza and grown in KGM and MEGM growth medium respectively. GM12878 cells were obtained from Coriell Institute for Medical Research and grown in RPMI 1640 media containing 2 mM L-glutamine, 10 % fetal bovine serum and 1 % penicillin/streptomycin.

### RNA extraction and quantitative Reverse-Transcriptase Polymerase Chain Reaction (qRT-PCR)

Total RNA was collected and purified, using the Direct-zol RNA Purification Kit (Zymo Research), from the following cell lines: BL30, RAJI, GM12878, NHEK, T47D, MCF7, UM-SCC-29, UM-SCC-103. Equal amounts of RNA (~1 μg) were reverse-transcribed to cDNA using the QuantiTect Reverse Transcription Kit (Qiagen). qRT-PCR assays were performed on the CFX96 Touch Real-Time PCR Detection System (Bio-Rad) using the iQ SYBR Green Supermix (Bio-Rad). Differential isoform specific gene expression was determined using the ΔΔCT method, using Glyceraldehyde 3-Phosphate Dehydrogenase (GAPDH) as an internal control. The expression levels are reported in terms of fold change relative to a specific cell type with the lowest levels of the two p63 isoform as determined by RNA-Seq data (RAJI for ΔNP63 and NHEK for TAp63). The following primers were used: TAp63 (Forward: CCCAGAGCACACAGACAAATG Reverse: GCGGATACAGTCCATGCTAATC), ΔNp63 (Forward: GAGCCAGAAGAAAGGACAGCAG Reverse: GAATCTGCTGGTCCATGCTGTTC), GAPDH (Forward: AGCCACATCGCTCAGACA Reverse: GCCCAATACGACCAAATCC).

### RNA-Seq analysis

Total RNA was extracted using the Direct-zol RNA MiniPrep kit (Zymo Research), from 6 HNSCC cell-lines: SCC15, SCC351, SCC4, SCC71, UM-SCC-29 and UM-SCC-103. For each RNA sample, cDNA libraries were prepared using the TrueSeq RNA Sample Preparation Kit (Illumina). The samples were then 50 bp single-end sequenced at ~ 25 million reads per sample on an Illumina HiSeq 2500. The RNA-Seq data has been deposited in GEO under the accession number GSE68872.

#### Analysis pipeline 1: TopHat, Cufflinks

Raw sequencing reads from the eight HNSCC RNA-Seq experiments (RNA-Seq for SCC15 and SCC351 cell-lines were done in replicates) and 46 publicly available datasets (22 cell-lines: 1 replicate, 12 cell-lines: 2 replicates) were mapped to the *Homo sapiens* genome *(hg19 build)* using TopHat (v2.0.7), with default parameters and Illumina’s iGenomes transcript annotation file ‘genes.gtf’ (from RefSeq; hg19) available at http://support.illumina.com/sequencing/sequencing_software/igenome.ilmn. Gene isoform level transcript abundances were quantified as Fragments Per Kilobase of transcript per Million mapped reads (FPKM) using Cufflinks (v2.1.1) [34].

#### Analysis pipeline 2: Bowtie, RSEM

Raw sequencing reads from the 54 experimental data sets were mapped to the *Homo sapiens* genome *(hg19 build)* using RSEM’s (v1.2.19) implementation of Bowtie (v1.1.1) [74] alignment program with poly-A option on and Illumina’s iGenomes transcript annotation file ‘genes.gtf’ (from Ensembl; GRCh37) (Script: rsem-prepare-reference). Gene and isoform level abundances were quantified as FPKM values using RSEM (Script: rsem-calculate-expression) [35].

### Clustering and network analysis

Transcript-probe level FPKM data as processed by Analysis Pipeline 1 from 40 cell-lines (54 experimental data points) was filtered for 1094 transcription factors (TFs) in *H.sapiens* (as annotated by QIAGEN’s Ingenuity Pathway Analysis: IPA, http://www.qiagen.com/ingenuity). We then performed a summation of expression for all possible transcripts of a TF to generate gene-probe level expression data. This was done for all 1093 TFs (excluding p63) resulting in a 1099 × 54 data matrix. This RNA-Seq expression matrix was then log transformed (log_2_ [FPKM + 1]). To reduce noise, we filtered the matrix to keep only significantly expressed (>5 FPKM in at least one experiment) and differentially expressed TFs (>2 fold difference between minimum and maximum expression values across all cell-lines). This final dataset (867 × 54) was then clustered using unsupervised hierarchical clustering based on average linkage and Pearson correlation distance metric as implemented in Cluster 3.0 software package [75]. The genes were centered on their median expression value across all experiments, prior to visualization of the clustering using Treeview [76]. ΔNp63 and TAp6-specific networks were generated using Ingenuity Pathway Analysis (IPA). The path explorer tool built the shortest relationship between ΔNp63 and TAp63 isoforms and their predicted transcriptional co-regulators. These relationships were then manually filtered to remove paths arising from pan-isoform studies, where no isoform specific function was provided. The networks were then overlaid with functions and disease data.

### Western blot

Western blot analysis was performed as previously described [77]. Primary antibodies were used at the following dilutions: Pan-p63 (Nekulova et al. [54], 1:5000), H-129 (Santa Cruz, 1:5000), TAp63 (Nekulova et al. [54], 1:5000), ΔNp63 (Nekulova et al. [54], 1:5000) and beta-Tubulin (Sigma, 1:10,000).

## Additional files

Additional file 1: Table S1. Cell-line database. Columns A–E characterize the 40 cell-types. Columns F–H provide information about the number of replicates, type of sequencing (single vs. paired end) and number of reads aligned. Column I give information about the p53 status of each of the cell-types (if known), as documented in the UMD TP53 mutation database (http://p53.free.fr/Database/p53_database.html). The PUBMED ID and reference for the studies that generated the RNA-Seq data for each of the cell-types is shown in columns J and K, respectively. (XLS 54 kb) Additional file 2: Figure S1. Expression estimates of genes are consistent across two independent analysis pipelines. RNA-Seq expression of 1094 TFs across 54 experiments was calculated by two methods: Analysis Pipeline 1 (Tophat, Cufflinks) and Analysis Pipeline 2 (Bowtie, RSEM). (A) Notched Box-plot depicting median correlation of 0.9 between RNA-Seq expression datasets (expression estimates in FPKM for 1094 TFs X 54 cell-lines) from the two methods. (B) Scatter plot with linear square fitted line showing high correlation between the expression estimates from the two methods (average across replicates), for 1094 TFs across individual representative cell-lines. (PDF 176 kb) Additional file 3: Table S2. Expression database. Column A contains the names of the 1099 TFs in humans. Columns B–BC provide the expression of the TFs in FPKM (fragments per kilobase of transcript per million), as calculated by Analysis Pipeline 1, across the 40 cell-types (52 experiments). (CSV 553 kb) Additional file 4: Figure S2 qRT-PCR reveals distinct expression patterns for TAp63 and ∆Np63. (A) Bar plot depicting normalized expression of ∆Np63 (to GAPDH) in a representative set of cell-lines. Asterix indicates that RAJI cell-line was used as reference control. (B) Bar plot depicting normalized expression of *T Ap63* (to GAPDH) in a representative set of cell-lines. Asterix indicates that NHEK cell-line was used as reference control. (PDF 45 kb) Additional file 5: Figure S3. ΔNp63α is the predominant isoform in both proliferating and differentiating keratinocytes. Line chart depicting expression of p63 isoforms in human keratinocytes at three time points during differentiation: Day 0(DK0), Day3 (DK3) and Day 6(DK6). ΔNp63α levels are attenuated during keratinocyte differentiation whereas TAp63 expression is not detectable at any time points under these conditions. Also shown are expression pattern of differentiation markers, FLG and EHF. FPKM: fragments per kilobase of transcript per million. (PDF 30 kb) Additional file 6: Figure S4. Chromatin landscape at the *TP63* gene locus in NHEK, GM12878 and MCF cells. A snapshot from the UCSC genome browser showing expression (RNA-Seq) and accessibility (DNase-Seq) at the *TP63* gene across three cell types: Normal Human Epidermal Keratinocytes (NHEK), B-lymphoblastoid (GM12878) and Breast adenocarcinoma (MCF7) cells. The dotted red boxes highlight the TA and ΔN promoters. (PDF 55 kb) Additional file 7: Figure S5. p63 isoform expression as estimated by Analysis Pipeline 2. Heatmap depicting the expression of p63 isoforms in FPKM (fragments per kilobase of transcript per million), across representative p63 expressing cell-lines. (PDF 44 kb) Additional file 8: Figure S6. Expression pattern of P53/P63/P73 family. Heatmap depicting the expression of p63 isoforms in relation to p53 and p73, across 40 human cell-types. These are commonly used cell-lines corresponding to all three germ layers and both normal and cancer karyotypes. Expression is quantified in FPKM (fragments per kilobase of transcript per million). (PDF 34 kb) Additional file 9: Figure S7. Unsupervised Clustering of the human cell-lines. The 40 cell-types (54 experiments) clustered using unsupervised hierarchical clustering based on average linkage and Pearson correlation distance metric. TAp63 expressing cell-lines are shown in green, ΔNp63 expressing cell-lines are shown in red. rep1: replicate 1, rep2: replicate 2. (PDF 30 kb) Additional file 10: Table S3. Biological functions enriched in ΔNp63 and TAp63 networks by IPA. Column A provides the diseases/biological functions, column B provides the *p* value of enrichment for the functions (calculated by Fisher’s exact test) and columns C and D provide the number and details of molecules that are involved in that function. (CSV 2 kb)
