# Supplementary figures and images for: A global analysis of the complex landscape of isoforms and regulatory networks of p63 in human cells and tissues
Source: BMC Genomics. 2015 Aug 7;16:584. doi: 10.1186/s12864-015-1793-9 (PMC4528692; doi:10.1186/s12864-015-1793-9)

**A**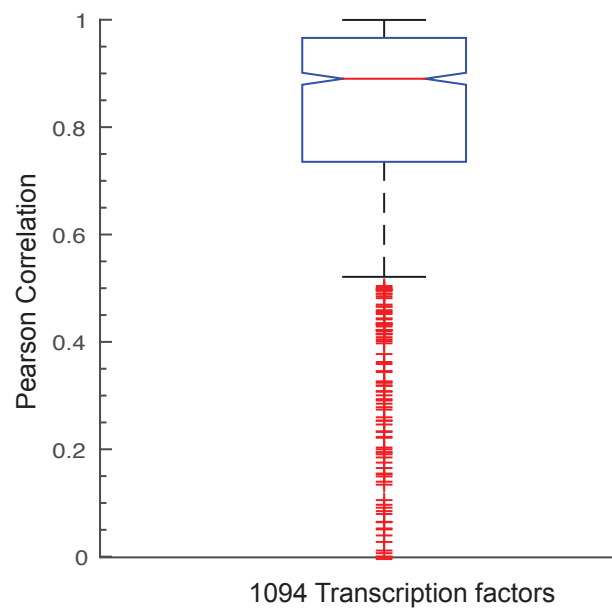**B**

SCC15

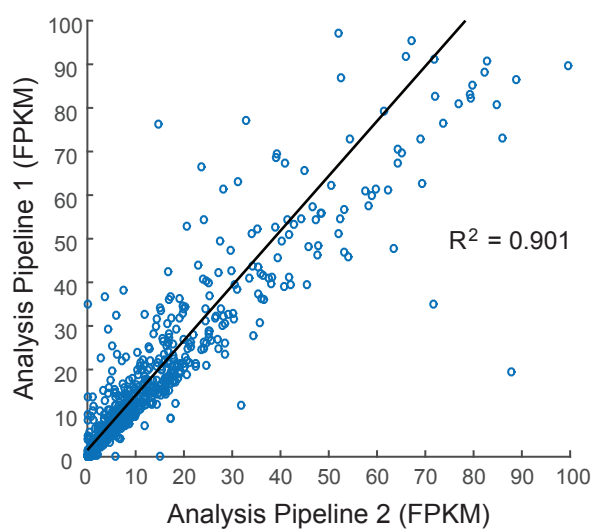

NHEK

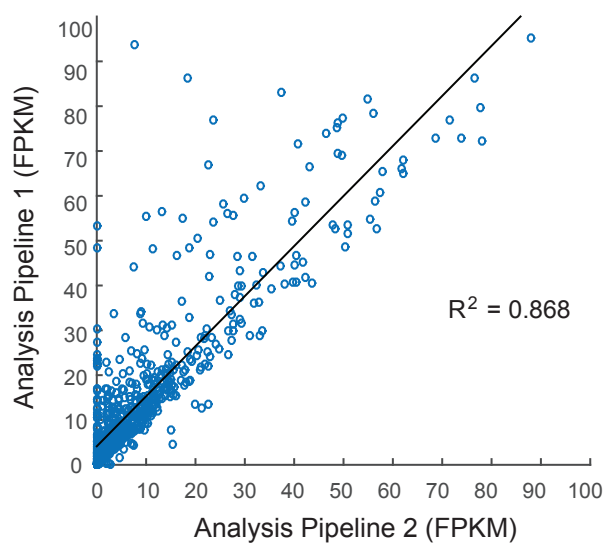

RAJI

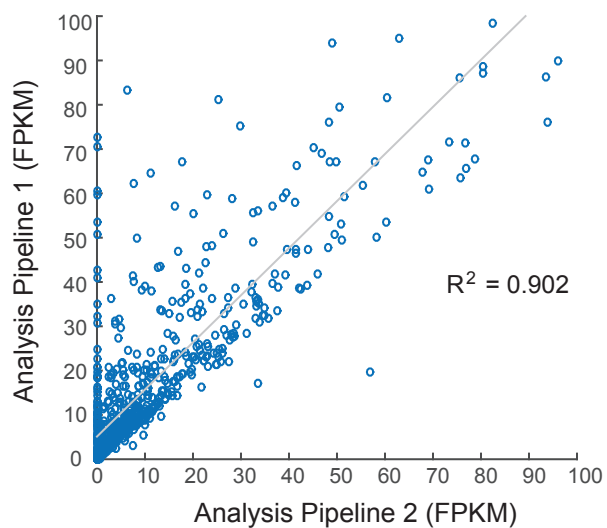

BL30

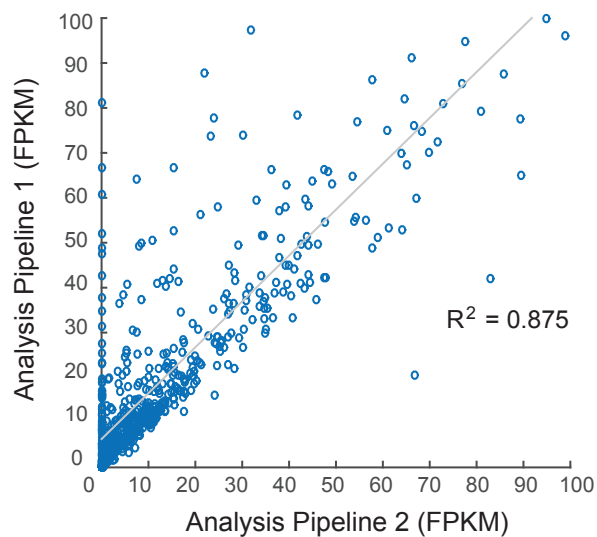

Supplement: Additional file 2: Figure S1. — Expression estimates of genes are consistent across two independent analysis pipelines. RNA-Seq expression of 1094 TFs across 54 experiments was calculated by two methods: Analysis Pipeline 1 (Tophat, Cufflinks) and Analysis Pipeline 2 (Bowtie, RSEM). (A) Notched Box-plot depicting median correlation of 0.9 between RNA-Seq expression datasets (expression estimates in FPKM for 1094 TFs X 54 cell-lines) from the two methods. (B) Scatter plot with linear square fitted line showing high correlation between the expression estimates from the two methods (average across replicates), for 1094 TFs across individual representative cell-lines. (PDF 176 kb) [file 12864_2015_1793_MOESM2_ESM.pdf]

**A**

# $\Delta$ Np63 Expression

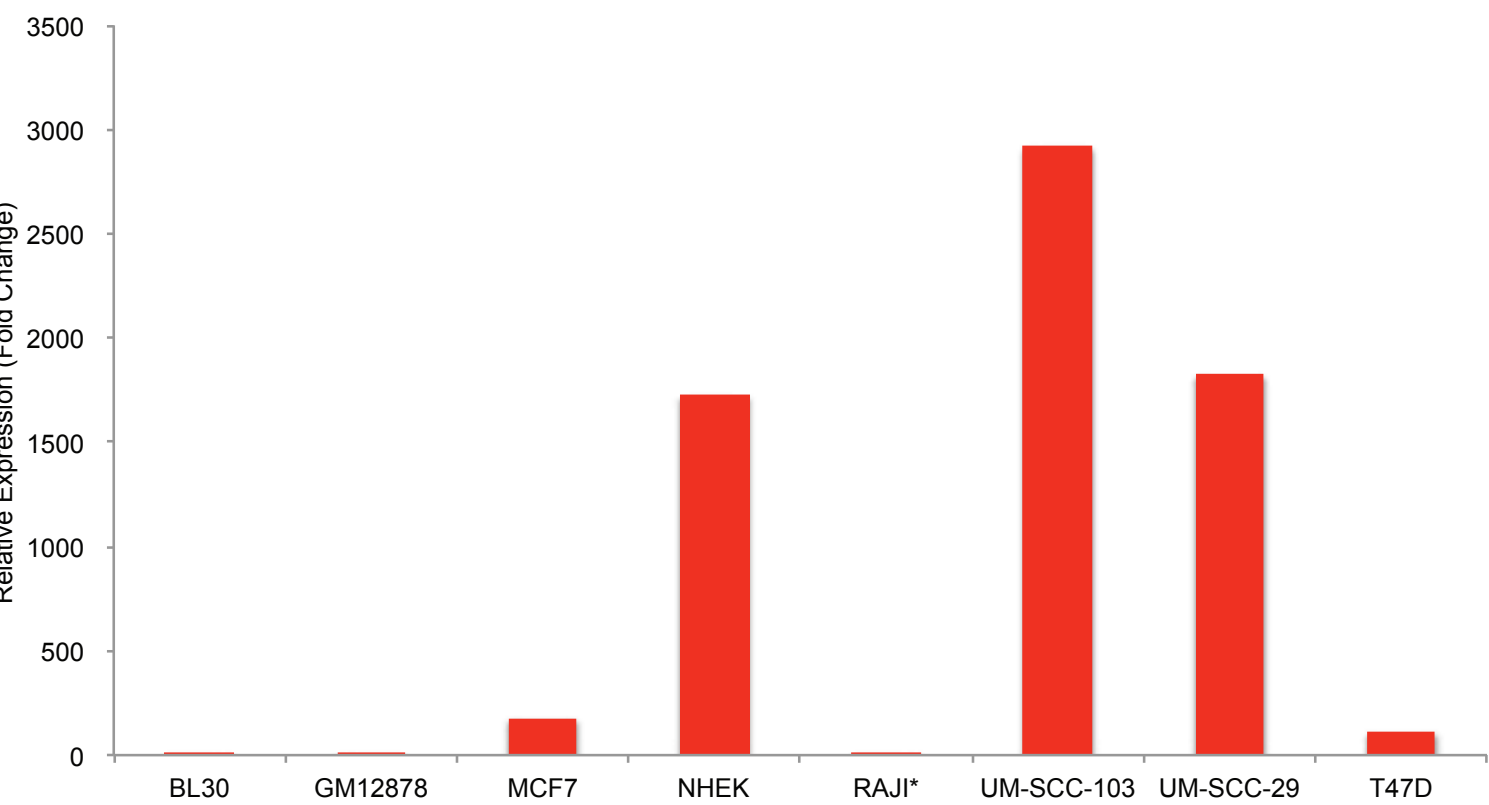

**B**

# TAp63 Expression

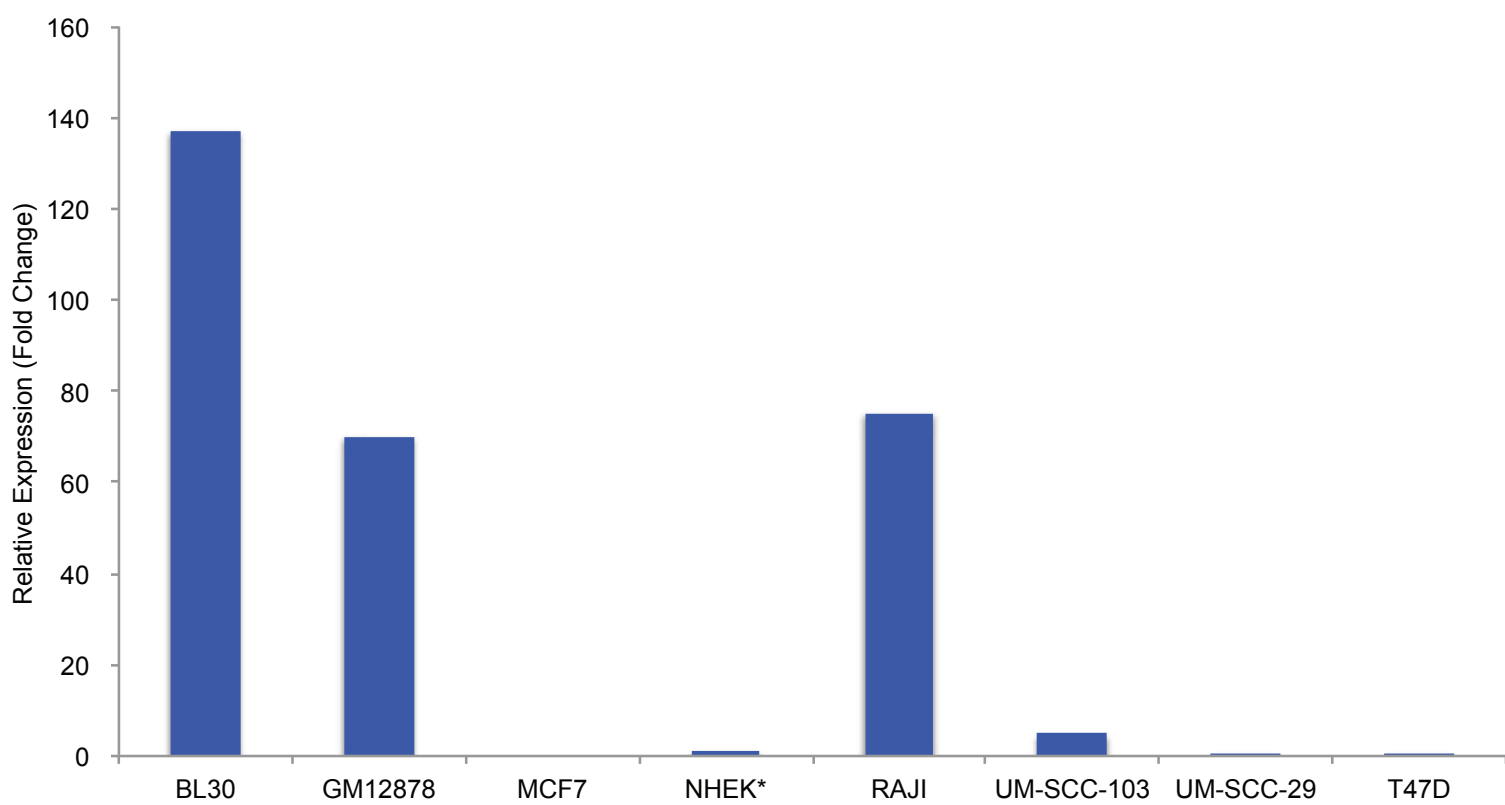

Supplement: Additional file 4: Figure S2 — qRT-PCR reveals distinct expression patterns for TAp63 and ∆Np63. (A) Bar plot depicting normalized expression of ∆Np63 (to GAPDH) in a representative set of cell-lines. Asterix indicates that RAJI cell-line was used as reference control. (B) Bar plot depicting normalized expression of T Ap63 (to GAPDH) in a representative set of cell-lines. Asterix indicates that NHEK cell-line was used as reference control. (PDF 45 kb) [file 12864_2015_1793_MOESM4_ESM.pdf]

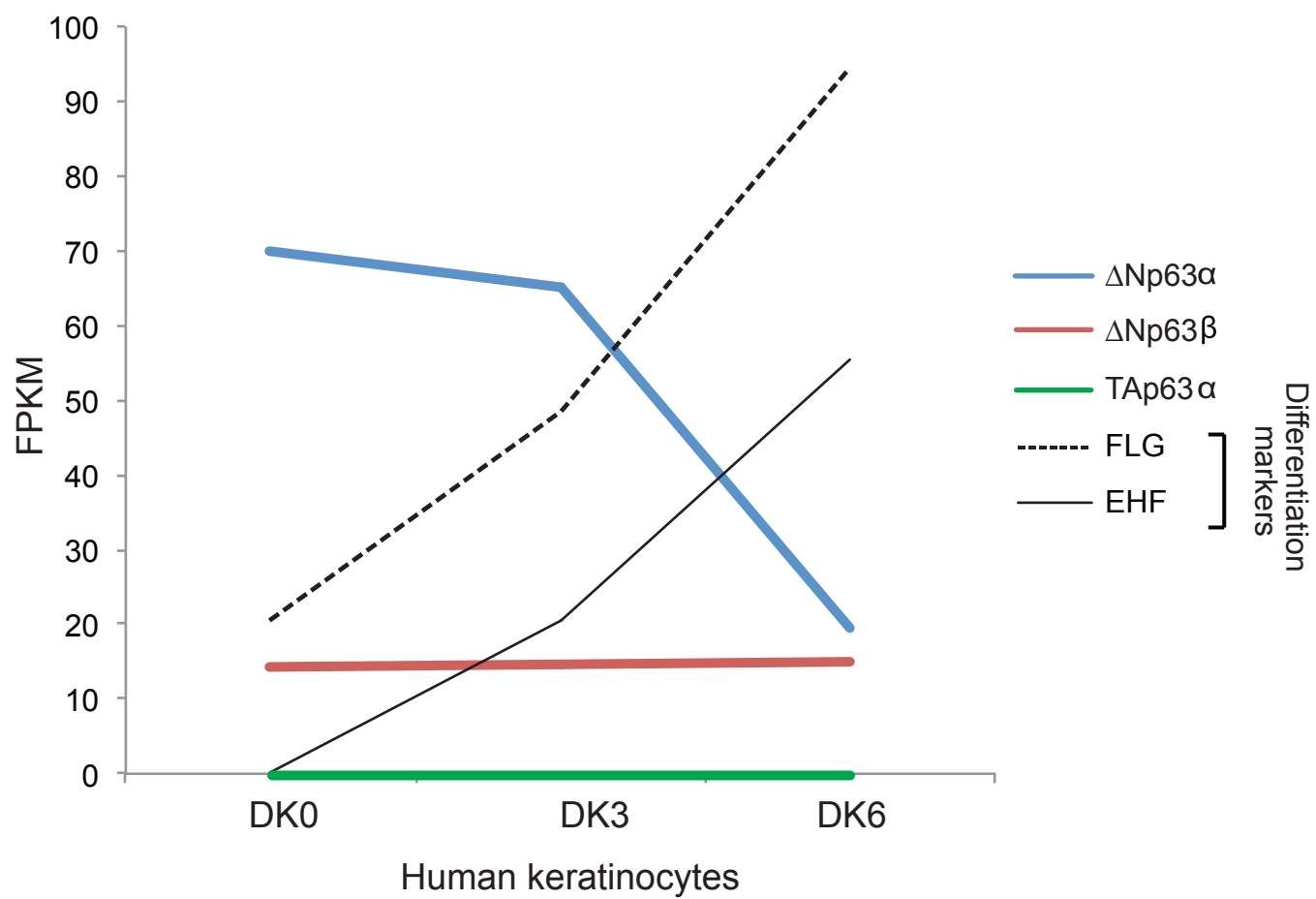

Supplement: Additional file 5: Figure S3. — ΔNp63α is the predominant isoform in both proliferating and differentiating keratinocytes. Line chart depicting expression of p63 isoforms in human keratinocytes at three time points during differentiation: Day 0(DK0), Day3 (DK3) and Day 6(DK6). ΔNp63α levels are attenuated during keratinocyte differentiation whereas TAp63 expression is not detectable at any time points under these conditions. Also shown are expression pattern of differentiation markers, FLG and EHF. FPKM: fragments per kilobase of transcript per million. (PDF 30 kb) [file 12864_2015_1793_MOESM5_ESM.pdf]

Cell-lines

Experiment

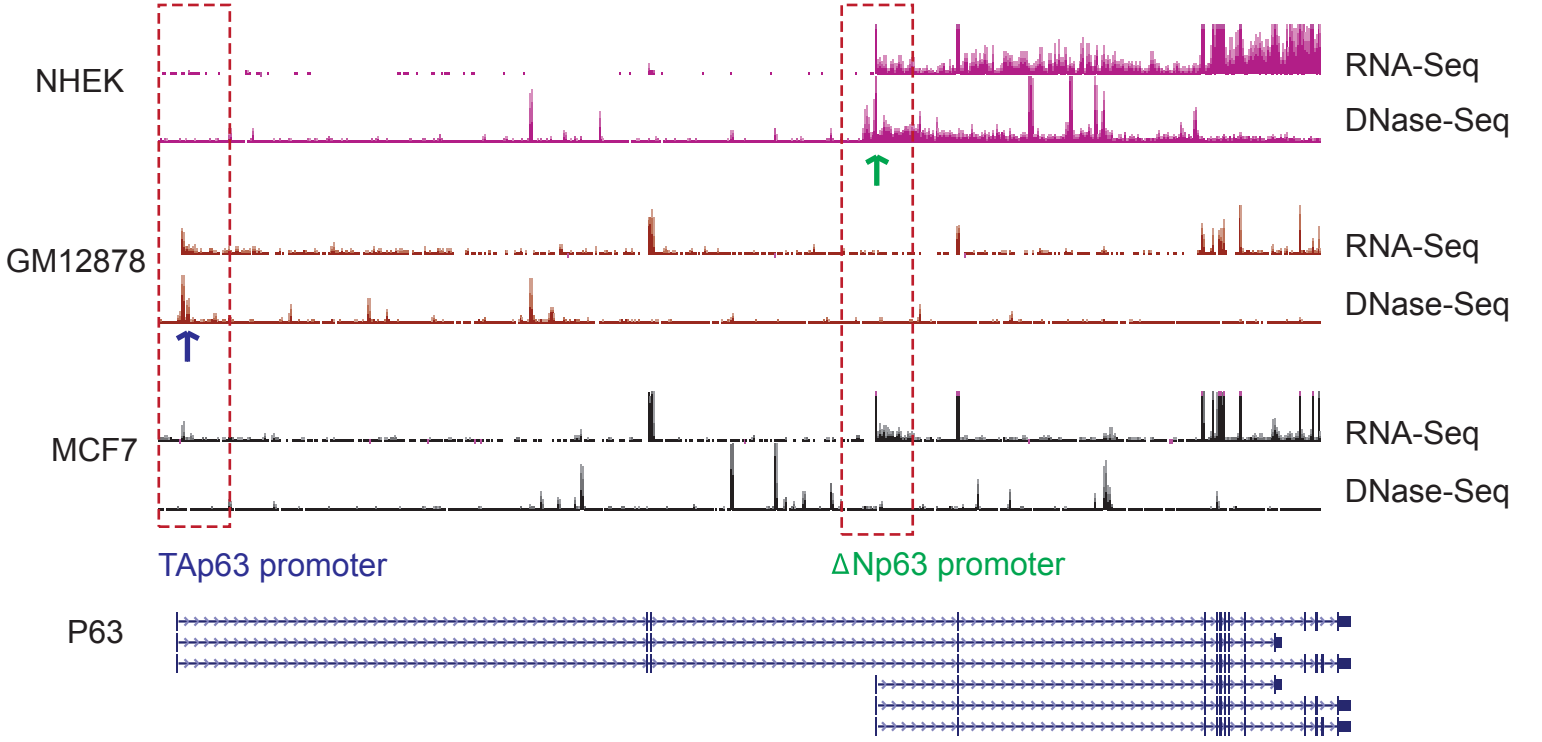

Supplement: Additional file 6: Figure S4. — Chromatin landscape at the TP63 gene locus in NHEK, GM12878 and MCF cells. A snapshot from the UCSC genome browser showing expression (RNA-Seq) and accessibility (DNase-Seq) at the TP63 gene across three cell types: Normal Human Epidermal Keratinocytes (NHEK), B-lymphoblastoid (GM12878) and Breast adenocarcinoma (MCF7) cells. The dotted red boxes highlight the TA and ΔN promoters. (PDF 55 kb) [file 12864_2015_1793_MOESM6_ESM.pdf]

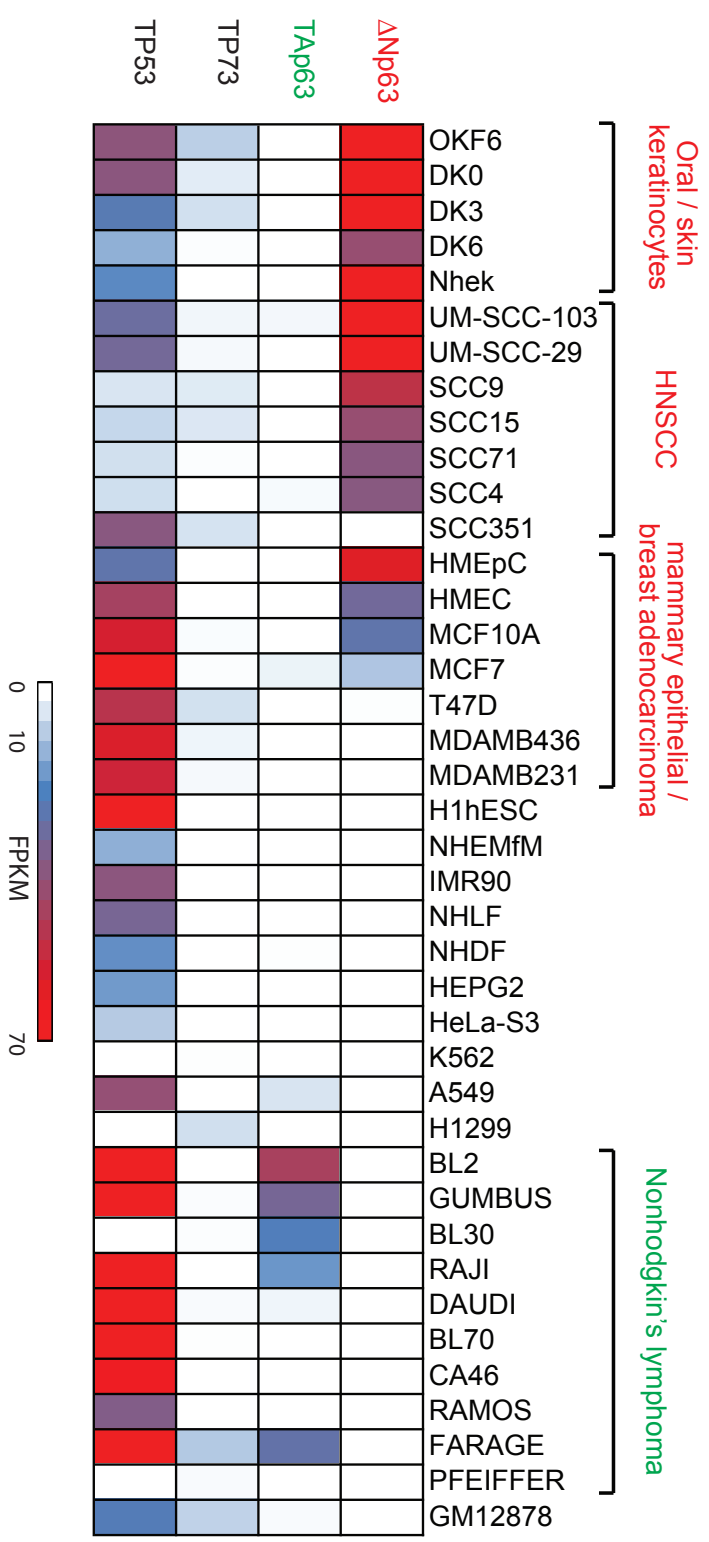

Supplement: Additional file 8: Figure S6. — Expression pattern of P53/P63/P73 family. Heatmap depicting the expression of p63 isoforms in relation to p53 and p73, across 40 human cell-types. These are commonly used cell-lines corresponding to all three germ layers and both normal and cancer karyotypes. Expression is quantified in FPKM (fragments per kilobase of transcript per million). (PDF 34 kb) [file 12864_2015_1793_MOESM8_ESM.pdf]

# Human Cell-lines

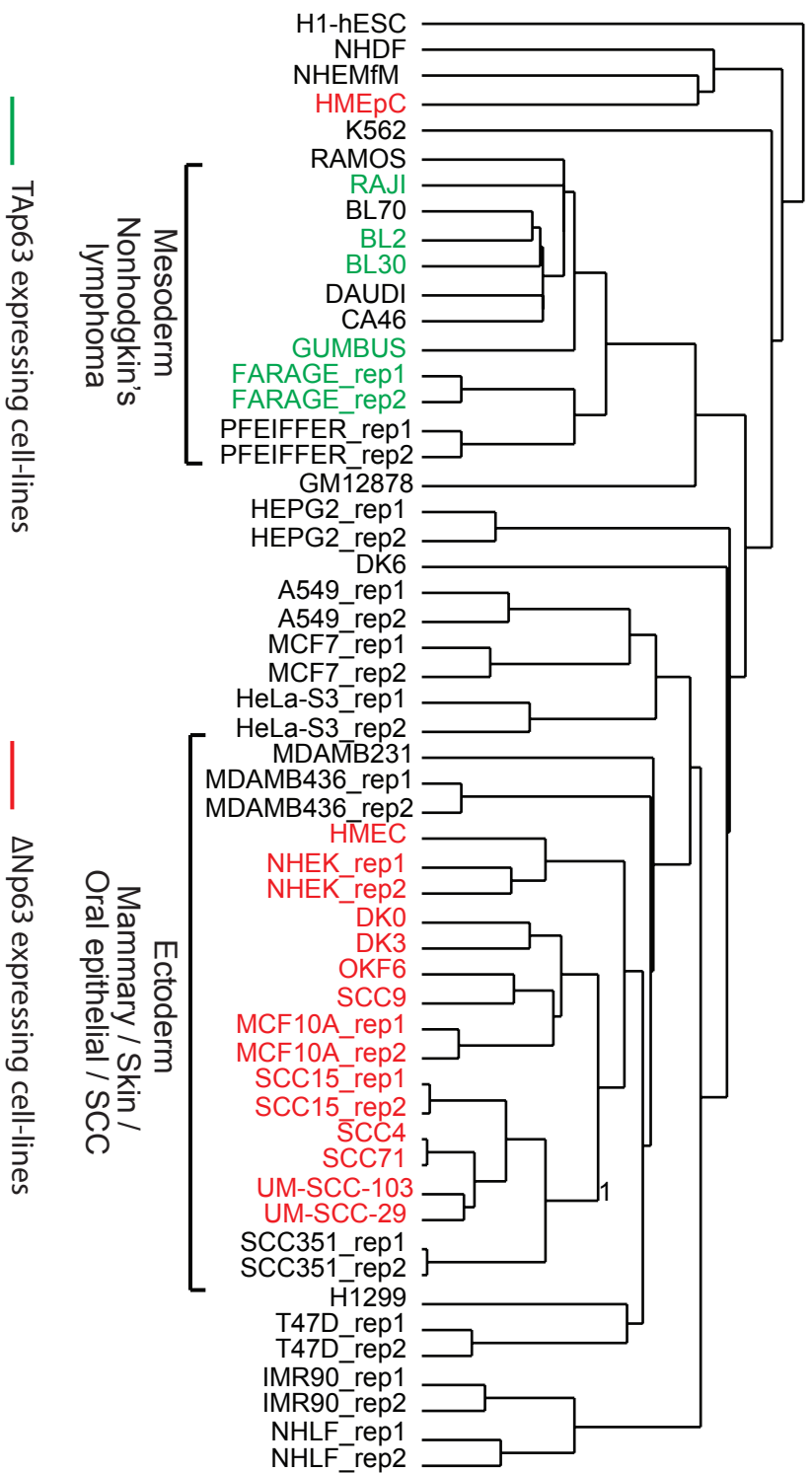

Supplement: Additional file 9: Figure S7. — Unsupervised Clustering of the human cell-lines. The 40 cell-types (54 experiments) clustered using unsupervised hierarchical clustering based on average linkage and Pearson correlation distance metric. TAp63 expressing cell-lines are shown in green, ΔNp63 expressing cell-lines are shown in red. rep1: replicate 1, rep2: replicate 2. (PDF 30 kb) [file 12864_2015_1793_MOESM9_ESM.pdf]
