# Supplementary material for: A global analysis of the complex landscape of isoforms and regulatory networks of p63 in human cells and tissues
Source: BMC Genomics. 2015 Aug 7;16:584. doi: 10.1186/s12864-015-1793-9 (PMC4528692; doi:10.1186/s12864-015-1793-9)
Supplement: Additional file 7: Figure S5. — p63 isoform expression as estimated by Analysis Pipeline 2. Heatmap depicting the expression of p63 isoforms in FPKM (fragments per kilobase of transcript per million), across representative p63 expressing cell-lines. (PDF 44 kb) [file 12864_2015_1793_MOESM7_ESM.pdf]

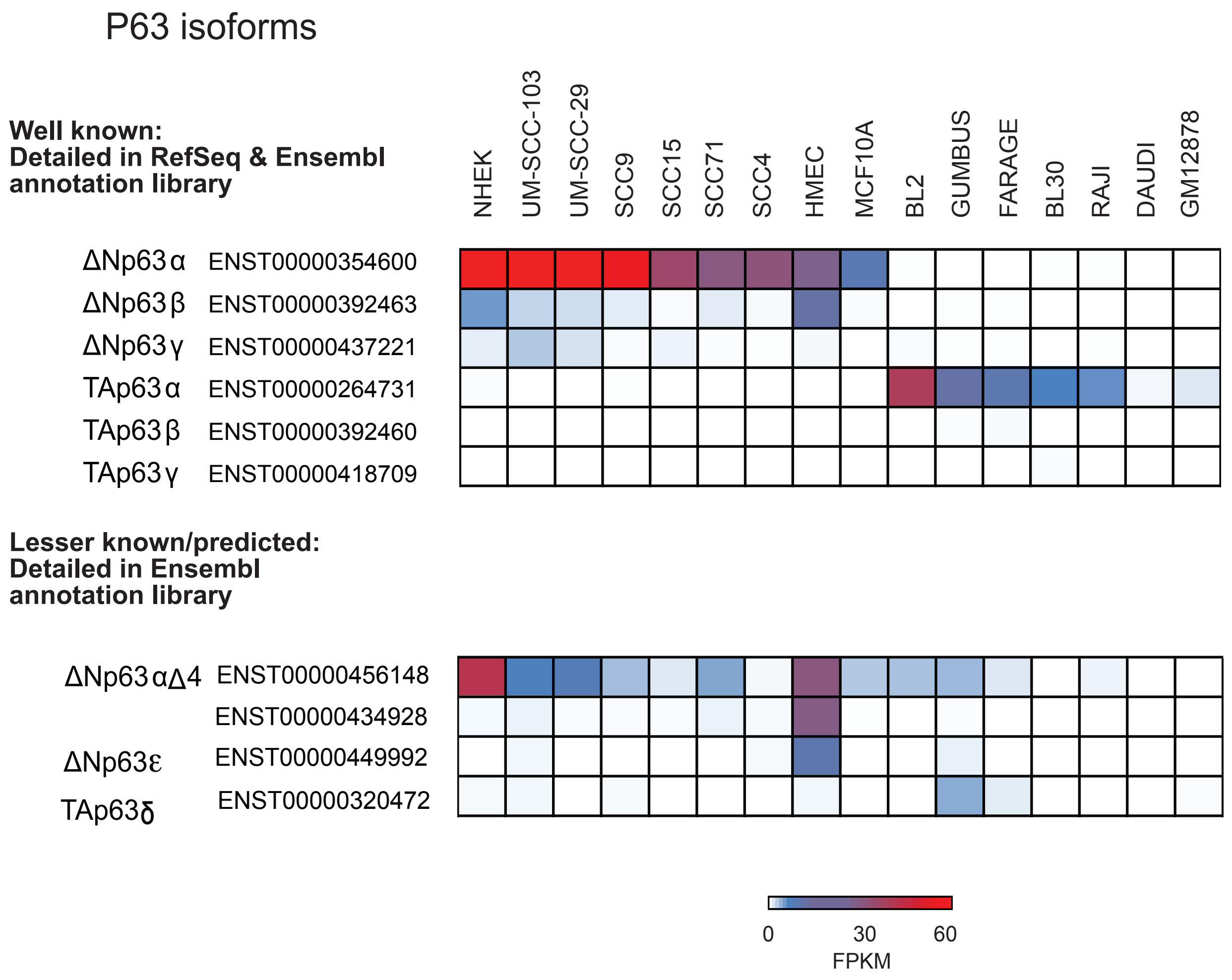
P63 isoforms
Well known:
Detailed in RefSeq & Ensembl
annotation library
NHEK
UM-SCC-103
UM-SCC-29
SCC9
SCC15
SCC71
SCC4
HMEC
MCF10A
BL2
GUMBUS
FARAGE
BL30
RAJI
DAUDI
GM12878
ΔNp63α ENST00000354600
ΔNp63β ENST00000392463
ΔNp63γ ENST00000437221
TAp63α ENST00000264731
TAp63β ENST00000392460
TAp63γ ENST00000418709
Lesser known/predicted:
Detailed in Ensembl
annotation library
ΔNp63αΔ4 ENST00000456148
ENST00000434928
ΔNp63ε ENST00000449992
TAp63δ ENST00000320472
0
30
60
FPKM
